# Management of arterial hypotension in critically Ill children: a narrative review and practical approach

**DOI:** 10.3389/fped.2026.1845899

**Published:** 2026-06-01

**Authors:** Hendryk Schneider

**Affiliations:** Center for Pediatrics, Division of Neonatology and Pediatric Intensive Care Medicine, Medical Center – University of Freiburg, Faculty of Medicine, University of Freiburg, Freiburg, Germany

**Keywords:** arterial hypotension, cardiac POCUS, hemodynamic monitoring, shock, vasoactive therapy

## Abstract

**Background:**

Arterial hypotension in critically ill children is a frequent and high-risk clinical finding, yet universally accepted definitions, thresholds, and management strategies remain lacking. Blood pressure is an accessible but imperfect surrogate for circulatory adequacy, and uncertainty persists regarding when and how aggressively hypotension should be treated.

**Objective:**

This narrative review aims to synthesize current evidence on the assessment and management of arterial hypotension in critically ill infants and children (beyond the neonatal period) and to translate these concepts into a structured, clinically applicable framework.

**Data sources and synthesis:**

This publication synthesizes current international guidelines, recent clinical studies, and expert consensus on pediatric hemodynamic monitoring and shock management. Particular emphasis is placed on the interpretation of blood pressure in context, age-dependent mean arterial pressure (MAP) targets, and the integration of clinical examination, laboratory parameters, and point-of-care echocardiography.

**Results:**

Arterial hypotension is typically a late sign of decompensated shock and is associated with increased mortality and adverse neurologic outcomes across multiple clinical scenarios, including septic shock, traumatic brain injury, and post-resuscitation care. MAP is the preferred parameter for assessment and therapeutic guidance. A pragmatic target of at least the 10th percentile for age appears reasonable in most critically ill children, balancing the risks of hypoperfusion and overtreatment. Early, repeated assessment using multimodal parameters—including cardiac point-of-care ultrasound—is essential. Initial management should prioritize rapid differentiation of shock etiology, judicious fluid resuscitation with balanced crystalloids, and early initiation of vasoactive therapy to avoid fluid overload. Emerging evidence supports norepinephrine as a first-line agent in distributive shock, with therapy tailored to underlying physiology.

**Conclusions:**

This review provides a pragmatic synthesis of current knowledge and presents a structured, evidence-based framework to support the bedside assessment and management of arterial hypotension in critically ill children. The inclusion of schematic approaches is intended to enhance clinical applicability by organizing existing evidence into an accessible format, while not representing original or unpublished data.

## Introduction

Blood pressure mathematically is the product of cardiac output (CO) and systemic vascular resistance (SVR). Therefore, repeated blood pressure readings provide important information about the hemodynamic status of the patients we treat. Low blood pressure occurs frequently in daily practice with ill children. Defining critical threshold values, the correct measurement method, interpretation in the context of the present disease and correct management vary between institutions and even between different providers of the same institution.

In fact, for critically ill children or children under anesthesia practicable consistent lower threshold values as well as management recomendations do not exist.

Moreover, regarding the necessity of treating arterial hypotension often there seems to be insecurity about the risk and benefits of initiating vasoactive therapy.

This review aims to synthesize current evidence into a structured and clinically applicable framework for the management of arterial hypotension in critically ill children (beyond the neonatal period >28 days of age).

Patients with congenital heart disease and those in the perioperative cardiac surgical setting require specific hemodynamic considerations and are therefore beyond the scope of this review.

Although this review focuses on arterial hypotension, management strategies are discussed within the broader context of circulatory failure, as myocardial dysfunction frequently contributes to hypotension and may require inotropic support.

All figures, tables, and the proposed clinical algorithm are derived exclusively from previously published studies, guidelines, and expert recommendations, and are intended as a conceptual synthesis of existing evidence rather than presentation of original or unpublished data.

## The rationale for managing arterial hypotension

The management of arterial hypotension in critically ill children is crucial because hypotension until proven otherwise is a late and ominous sign of shock, indicating failure of compensatory mechanisms, imminent risk of inadequate tissue perfusion and organ dysfunction. A low blood pressure on admission is related to an increased mortality (1). Shock itself, and particularly septic and hypovolemic shock, is one of the leading causes of morbidity and mortality in children worldwide (2, 3). Early recognition and intervention are essential to prevent progression to decompensated shock, which is associated with increased morbidity and mortality (4).

Essentially managing arterial hypotension is all about recognizing and reversing the state of decompensated shock. Shock itself is defined by the failure of oxygen delivery to meet tissue metabolic demands, and hypotension marks the transition to decompensated shock, often accompanied by signs of end-organ hypoperfusion such as altered mental status, oliguria, and acidosis (5).

Oxygen delivery is the product of cardiac output (CO) and arterial oxygen content (CaO_2_). Blood pressure is only one of few surrogate parameters for CO, not a direct measure (6). Cardiac output may be maintained by compensatory mechanisms even when blood pressure is low, but once hypotension occurs, cardiac output is usually critically reduced, and end-organ perfusion and consequently oxygen delivery is threatened. Thus, blood pressure alone is insufficient to assess circulatory adequacy, but persistent hypotension is a marker of inadequate cardiac output and consequently oxygen delivery (7).

In post-resuscitation care, both the presence and severity of post-arrest hypotension are associated with lower survival rates and worse neurologic outcomes in children. Maintaining blood pressure above age-specific thresholds improves survival and neurologic outcomes after cardiac arrest (8, 9).

In pediatric traumatic brain injury (TBI), hypotension is strongly associated with increased mortality and poor neurologic outcomes (10).

Moreover, arterial hypotension is a major risk factor for failed or complicated intubation, including peri-intubation cardiac arrest, in critically ill children. Hypotensive children have higher rates of peri-intubation arrest, lower first-pass success, and increased mortality (11, 12).

Lastly, it has been described that children, particularly infants, can develop signs of hypoxic ischemic encephalopathy following general anesthesia in the context of intraoperative arterial hypotension (13). Although sustained hypotension is associated with increased risk of poor neurological outcome, large multicenter observational studies have shown that intraoperative hypotension is common in neonates and infants, especially after induction and during prolonged procedures. Still, universally accepted lower limits of mean arterial pressure for anesthetized infants remain undefined (14, 15).

Although continuous cardiac output monitoring in critically ill children would be desirable, the evidence and recommendations concerning this matter are based on expert opinion due to a lack of high-quality outcome data. Recent reviews highlight that while invasive techniques such as transpulmonary thermodilution and pulmonary artery catheterization provide the most reliable continuous cardiac output data, their use is generally reserved for the most severe or complex cases due to procedural risks and lack of demonstrated outcome benefit in pediatric populations (7). Noninvasive and minimally invasive cardiac output monitors (e.g., pulse contour analysis, electrical cardiometry) are less reliable in unstable or rapidly changing hemodynamic states, though if available they may be useful adjuncts in stable patients or for trending purposes (16–18).

In summary there is no single clinical parameter that allows to evaluate the global hemodynamic status in children. Therefore, it is recommended to analyze several parameters by frequent assessments. These parameters include heart rate, capillary refill time, state of consciousness, lactate measurement, central venous oxygen saturation (ScvO2 > 70%), urine output as well as point of care echocardiography and particularly blood pressure (4, 7, 19, 20).

Blood pressure in the end is one of these parameters that is accessable, enables continuous data sampling and for which comparable thresholds for intervention can easily be defined in clinical practice, which in turn seems to influence survival and neurologic outcome in critically ill, as well as anesthetized children (8, 10, 21, 22). Moreover, blood pressure is the most used clinical assessment parameter (93%) of the panel members of the current Surviving Sepsis Campaign International Guidelines for the Management of Sepsis and Septic Shock in Children 2026 (19).

*Synopsis:*

**Arterial hypotension** in critically ill children until proven otherwise is a late and ominous sign of **shock**, indicating failure of compensatory mechanisms and imminent risk of inadequate tissue perfusion and organ dysfunction.
  - In **post-resuscitation** care, both the presence and severity of post-arrest hypotension are associated with **lower survival rates** and **worse neurologic outcomes** in children.
  - *In **TBI** arterial hypotension is associated with **increased mortality** and **poor neurological outcome.***
  - *Arterial hypotension is a major **risk factor** for **failed or complicated intubation**, including peri-intubation cardiac arrest, in critically ill children.*
  - *Intraoperative arterial hypotension is a risk factor for **hypoxic ischemic encephalopathy**.*

## Invasive vs. Non-invasive blood pressure—mean arterial pressure vs. systolic blood pressure

During the initial evaluation of critically ill children non-invasive (usually oscillometric) blood pressure measurement is widely used due to its ease of use, safety and ability to provide rapid readings.

Intra-arterial (invasive) measurement, typically via an indwelling arterial catheter, provides continuous, beat-to-beat blood pressure data and is considered the gold standard for accuracy, especially in critically ill children. Oscillometric devices tend to overestimate MAP during hypotension and underestimate it during hypertension, and their accuracy decreases in neonates, infants, and during extremes of blood pressure (23–25).

Although invasive measurement provides the more accurate data and offers the possibility to take arterial blood samples, it is more technically challenging, carries the risks of vascular injury, thrombosis and infection, and is therefore less feasible for routine monitoring, especially in the initial stabilization phase.

Although, major complications of arterial lines in children seem to be rare, with an overall incidence of 0.2% (2 per 1,000 lines) (26), the benefits should still outweigh the risks.

A higher catheter-to-artery diameter ratio is strongly associated with immediate complications, including thrombus formation and arterial stenosis, after radial arterial catheterization in young children (odds ratio 25.3, 95% CI 1.2–350.7) (27).

Ultrasound guidance for arterial line placement improves first-attempt, overall success rate, reduces procedure time and number of puncture attempts, and furthermore lowers complication rate in comparison to traditional methods (28).

Routine continuous UFH infusion does not reliably reduce thrombosis or vascular injury rates for arterial lines in critically ill children in pediatric intensive care units (29), unlike the reduction observed with bolus UFH during arterial catheterization (30).

Conversely, data from a recent study in adult patients with shock suggest that management without early arterial catheter insertion was noninferior to early catheter insertion (31), which could also challenge the practice of early arterial catheterization in children with arterial hypotension due to shock.

Mean arterial pressure is the most accurate value in non-invasive oscillometric measurement and should be used preferentially for assessing hypotension in children (24).

One rationale for this is the method of oscillometry—Oscillometric blood pressure measurement works by detecting pressure oscillations in an inflating/deflating cuff, identifying the point of maximum oscillation (which is the mean arterial pressure), and mathematically estimating systolic and diastolic pressures from the oscillation pattern (32).

Secondly, for non-invasive and invasive blood pressure measurements several guidelines reasonably recommend targeting mean arterial pressure values:

The Surviving Sepsis Campaign recommends targeting mean arterial pressure (19).

For children with traumatic brain injury (TBI) and/or increased intracranial pressure (ICP) targeting mean arterial pressure (MAP) is physiologically justified, as it reflects the driving pressure for organ perfusion, particularly cerebral perfusion pressure (CPP = MAP – ICP) (10, 33).

For children following resuscitation targeting MAP or systolic blood pressure (SBP) is recommended (8, 21, 22), although of course because of the risk of cerebral edema and increased ICP defining target values for the MAP is more reasonable (10, 33).

Another important argument for universally using the MAP to guide hemodynamic monitoring and management in critically ill children is, that hyperdynamic states, such as in the initial phase of distributive shock (sepsis, systemic inflammatory response syndrome or cytokine release syndrome) SBP may remain within normal limits or even be elevated due to increased stroke volume, while MAP and diastolic blood pressure (DBP) are reduced, resulting in compromised organ perfusion despite apparently adequate SBP (4, 34).

Lastly age-based percentile curves for MAP are available and therefore can easily be used to guide therapy (35).

*Synopsis:*

In the initial stabilization phase of critically ill children it is reasonable to use **repeated non-invasive (oscillometric) measurement.**
Placing an **arterial line** (using the **smallest possible catheter size and sonography** for placement) should be considered in patients with hemodynamic instability.
Using the **mean arterial pressure** (rather than the systolic blood pressure) to guide therapy for non-invasive as well as invasive measurements is a pragmatic approach.

## Defining mean arterial pressure treatment thresholds

There is substantial variability in the definitions and intervention thresholds for arterial hypotension in pediatric intensive care, both across clinical guidelines and population-based centile charts. Clinical guidelines often use stepwise age-based cutoffs or formulas, but these show low to moderate agreement with population-based lower centiles, and thresholds can differ by as much as 15–30 mmHg depending on the guideline and age group (36–38). Importantly, thresholds derived from healthy children may not be appropriate for critically ill populations, as these children often have altered physiology and increased vulnerability to hypoperfusion (37, 38).

Percentile-based thresholds for SBP and MAP have been proposed to provide more individualized targets. For example, formulas such as SBP (5th percentile at 50th height percentile) = 2 × age in years + 65 and MAP (5th percentile at 50th height percentile) = 1.5 × age in years + 40 are derived from population data (37).

Observational studies of intraoperative hypotension show that intervention thresholds for MAP increase with age, with median values ranging from 36 mmHg in infants to 57 mmHg in teenagers (39).

The Surviving Sepsis Campaign recommends targeting mean arterial pressure between the 5th and 50th percentile for age in pediatric septic shock (19).

For children with TBI and/or ICP, CPP depends on MAP (CPP = MAP − ICP), and maintaining age-appropriate MAP is essential for adequate cerebral perfusion. Pediatric consensus guidelines recommend CPP targets above 40 mmHg for children aged 0–5 years and above 50 mmHg for those aged 6–17 years, with MAP adjusted accordingly to maintain these CPP goals (33). MAP <10th percentile during the first 12 h after pediatric intensive care unit (PICU) admission was associated with a higher risk of poor discharge outcome in children with severe TBI when compared to children whose lowest MAP percentile was between the 50th and 94th percentile (10).

The recommended blood pressure target for children following resuscitation is to maintain MAP greater than the 10th percentile for age, because it is associated with increased survival to hospital discharge and survival with favorable neurologic outcome. This threshold is supported by the American Heart Association and American Academy of Pediatrics guidelines, which also recommend continuous arterial pressure monitoring when resources allow (8, 21, 22).

On the other hand in pediatric septic shock, a single-center randomized controlled trial (RCT) found that targeting the 5th vs. 50th percentile for MBP resulted in similar mortality, but lower vasoactive drug use and fewer adverse events in the lower target group, suggesting that a lower threshold may be safe and reduce overtreatment (40). These findings highlight the ongoing uncertainty regarding optimal MAP targets in pediatric septic shock and support a more individualized approach, particularly in light of emerging concepts of permissive hypotension.

The PRESSURE trial (designed as pragmatic, open, multicenter, parallel-group RCT) tries to evaluate a permissive MAP target greater than the 5th centile for age vs. usual care in critically ill children, using a composite outcome of mortality and duration of invasive mechanical ventilation from randomization to day 30 (41).

A evidence-informed, clinically applicable approach is to use coherent age-based MAP percentiles by Roberts et al. (35) and aim at the 10th to 20th percentile for pediatric ICU patients (Table 1), because this is supported by guideline recommendations to maintain MAP above the 10th percentile for age, and outcome studies associating MAP below the 10th percentile with increased risk of mortality and unfavorable neurologic outcomes (5, 10, 21). Targeting the 10th–20th percentile may provide a safety margin above the lowest normal values, balancing the risk of hypoperfusion with avoidance of unnecessary vasoactive exposure while meeting suggested aims with a security margin. Although, MAP targets may differ depending on the clinical context (5th percentile in children with sepsis vs. 10th percentile in post-cardiac arrest patients).

**Table 1:** Mean arterial pressure (MAP) target values for PICU patients based on published guidelines and studies (8, 10, 19, 22, 33, 35).

Age	MAP (Min.-Max.) [mmHg]
Term neonate (≤ 7 days of life)	40–70
Newborn (8–28 days of life)	45–80
Infant (2–12 months of life)	50–85
13–24 months of life	55–95
3–6 years of life	57–90
7–12 years of life	60–90
>12 years of life	65–90

Consistent use of clear lower blood pressure limits in the PICU may help to facilitate timely recognition and intervention for hypotension, potentially improving outcomes and standardizing care.

Ongoing research, including multicenter RCTs, are needed to refine optimal intervention thresholds and link them to patient-centered outcomes.

*Synopsis:*

Define age-dependent intervention thresholds for mean arterial pressure values (also see Table 1).
It is reasonable to aim at least for the **10th percentile for age** according to recommendations for children with TBI, in the post-resuscitation setting as well as those with distributive (septic) shock.

### It is not always shock!

Oversedation is more common than undersedation in the PICU, and optimal sedation practices—including regular assessment and minimization of sedative exposure—are critical to reducing adverse hemodynamic events (42, 43). Oversedation, regardless of agent, increases the risk of hypotension, prolonged mechanical ventilation, and PICU complications like delirium, highlighting the importance of titrating sedation to effect and using validated assessment tools (43, 44). Thus, the possibility of oversedation in critically ill children, especially those with sedation and neuromuscular blockade should be taken into account and reduction of sedation, the use of processed electroencephalography (pEEG) monitors, as well as neuromuscular blockade (NMB) discontinuation should be disscussed (45).

In addition, other iatrogenic causes of hypotension should be considered, including the effects of vasoactive or cardiovascular medications such as milrinone, levosimendan or angiotensin-converting enzyme inhibitors, which may lead to intended or unintended reductions in cardiac afterload and blood pressure (46–49).

Furthermore, studies demonstrate that incorrect blood pressure cuff sizes—regardless of index lines—can result in significant over- or underestimation of blood pressure, with smaller cuffs causing falsely elevated readings and larger cuffs causing falsely low readings (50). The American Heart Association emphasizes that accurate pediatric blood pressure measurement requires selecting a cuff with a bladder width at least 40% of the mid-arm circumference and a length covering 80%–100% of the arm, determined by direct measurement rather than relying on manufacturer markings or index lines, which are not standardized and can be misleading across brands (51).

*Synopsis:*

In sedated children arterial hypotension not uncommonly is due to **oversedation**—monitoring of sedation depth (using designated scores and/or pEEG), and probatory reduction of sedation agents are recommended.
If NMB is used, pEEG monitoring and/or NMB discontinuation should be considered if applicable.
**Vasoactive and cardiovascular medications** may cause intended or unintended arterial hypotension.
Use **appropriate cuff sizes** in non-invasive blood pressure measurement (cuff bladder length 80% and a width 40% of arm circumference).

## Arterial hypotension due to shock

While definitions and thresholds for hypotension in children vary, arterial hypotension in children consistently shows high specificity (but low sensitivity) for serious illness and ICU admission (36, 38, 52), and is until proven otherwise a sign for decompensated shock.

Shock in children is defined as the failure of oxygen delivery to meet tissue metabolic demands and is life-threatening. The American Heart Association and American Academy of Pediatrics categorize pediatric shock into four major types: hypovolemic, distributive (including septic), cardiogenic, and obstructive (Table 2). Multiple types may coexist, and early presentations can be subtle, requiring vigilance for signs such as tachycardia, decreased peripheral perfusion, and later hypotension with end-organ dysfunction (5, 53).

**Table 2:** Categories of shock (53).

Category	Hemodynamics	Causes
Hypovolemic	Preload ↓, SVR↑, CO↓	Gastrointestinal loses, Renal loses, Hemorrhage, Third spacing/capillary leak
Distributive	Preload ↓, SVR↓↓, CO↓↑	Sepsis/SIRS, Anaphylaxis, Neurogenic shock
Cardiogenic	Preload ↑, SVR↑, CO↓	Congenital heart disease, Arrhythmia, Cardiomyopathy, Myocarditis, Severe anemia
Obstructive	Preload ↑↓, SVR↑, CO↓	Pulmonary embolism/(severe) pulmonary hypertension, Pericardial tamponade, Tension pneumothorax, Shunt dependent congenital heart lesions

CO, cardiac output; SVR, systemic vascular resistance; SIRS, systemic inflammatory response syndrome.

The presence of shock is also associated with worse outcomes in a variety of emergency conditions, including traumatic brain injury and cardiac arrest (9, 54).

Correct categorization of shock is essential for guiding therapy. Hypovolemic shock is managed primarily with fluid resuscitation, while distributive shock (e.g., septic shock) requires fluids and vasopressors. Cardiogenic shock necessitates inotropes and careful fluid management, and obstructive shock requires immediate relief of the obstruction. The American Heart Association and American Academy of Pediatrics emphasize that therapy should be tailored to the underlying physiology, and inappropriate therapy (e.g., excessive fluids in cardiogenic shock) can worsen outcomes (5). However, clinical signs used to categorize shock (e.g., extremity temperature, capillary refill) have low inter-rater agreement, and mismatch between shock type and vasoactive choice is can be associated with worse outcomes, suggesting caution in relying solely on clinical categorization (55). Furthermore, overlap between different types of shock is common in pediatric patients, and this is well recognized in both clinical guidelines and observational studies. Early recognition of mixed shock states is critical for appropriate management and improved outcomes (5).

The distribution of the types of shock in children unfortunately is not well studied. Retrospective data suggests the distributive, especially septic shock accounts for the majority of cases, with hypovolemic shock (often from gastroenteritis or hemorrhage) as the next most frequent form (56).

Most guidelines are concentrating on septic shock, as a form of distributive shock. In daily work we often handle patients with suspected septic shock or SIRS and/or capillary leak.

Importantly, from a clinicians standpoint a lot of recommendations for septic and septic shock patients may very well transferrable to patients with distributive shock including those with severe capillary leak and/or systemic inflammatory response syndrome (post-surgical patients, polytrauma patients, patients with severe hemorrhagic shock, post rescucitation care patients or children with cytokine release syndrome) because the physiology is very comparable.

Furthermore, acute pulmonary hypertension may complicate the course of various cardiovascular, pulmonary, and systemic conditions, including congenital heart disease, chronic parenchymal lung disease (e.g., bronchopulmonary dysplasia), acute lung diseases (e.g., pneumonia and pediatric acute respiratory distress syndrome), as well as sepsis. In such scenarios, pulmonary hypertension can contribute to systemic hypotension, as increased pulmonary vascular resistance may impair right ventricular output and reduce left ventricular preload, ultimately leading to decreased cardiac output. This may reflect features of both obstructive and cardiogenic shock physiology (57).

Pericardial tamponade is classically categorized as a form of obstructive shock, as impaired cardiac filling due to increased intrapericardial pressure leads to reduced preload and cardiac output. From a hemodynamic perspective, this may present with features resembling cardiogenic shock, highlighting the importance of integrating clinical and ultrasound findings.

The available tools for the treatment of distributive, hypovolemic and cardiogenic shock with or without arterial hypotension include volume therapy, inotropic and vasoactive agents, hydrocortisone, blood products, and extracorporeal life support if applicable.

Most importantly, management of arterial hypotension in shock must always include identification and treatment of the underlying cause. While hemodynamic stabilization is essential, definitive therapy—such as antimicrobial treatment in sepsis, source control, or relief of obstructive processes—remains the cornerstone of effective management.

*Synopsis:*

Arterial hypotension in children shows high specificity for serious illness, since it is until proven otherwise a sign for **decompensated shock**.
Correct categorization of shock (hypovolemic, distributive, cardiogenic, obstructive) is essential for guiding therapy.
Timely **identification and treatment** of the underlying **cause** of shock, in parallel with hemodynamic stabilization, remain the cornerstone of management.

## Reasonable fluid management

Recent clinical trials and systematic reviews reinforce that balanced or buffered crystalloid solutions are preferred over normal saline for initial resuscitation in pediatric shock, as they are associated with a lower risk of acute kidney injury and hyperchloremia, though mortality outcomes are similar between fluid types (19). Colloid solutions, including albumin, do not confer a clear benefit over crystalloids and are not recommended as first-line therapy due to cost and potential nephrotoxicity (4, 58, 59). Nevertheless, a recent secondary analysis of electronic health record data from 13 U.S. PICUs showed that early albumin administration was associated with lower in hospital-mortality in children with septic shock. Though prospective validation is warranted (60).

Peripheral venous access remains the preferred route for initial fluid administration, with intraosseous access as a rapid and effective alternative when peripheral access is not immediately available. Central venous access is not recommended for initial resuscitation unless already in place or if experienced personnel can place it quickly; it is more useful for ongoing hemodynamic monitoring and vasoactive drug administration (61).

Volume therapy can easily lead to fluid overload. Fluid overload itself can be hazardous, particularly for children with septic shock, cerebral edema, or risk of abdominal compartment syndrome. Randomized trials and observational studies show that higher fluid volumes or faster bolus administration increase rates of mechanical ventilation and worsen oxygenation, underscoring the need for frequent reassessment and early discontinuation of fluids if signs of overload develop (5, 62).

Observational studies, meta-analyses, and international guidelines demonstrate that fluid overload—defined as cumulative positive fluid balance ≥5%–10% of body weight—within the first days of PICU admission is independently associated with increased mortality, prolonged mechanical ventilation, acute kidney injury, and longer PICU stays in pediatric septic shock, even after adjusting for illness severity and comorbidities (63, 64).

Echocardiography is increasingly recognized as a key tool for guiding fluid therapy. It enables real-time assessment of cardiac function, preload responsiveness, and detection of myocardial dysfunction, and is valuable for monitoring the adequacy of resuscitation and identifying patients at risk for fluid overload or requiring de-escalation of therapy (65, 66).

Early use of vasoactive agents can help in preventing fluid overload in critically ill children with hypotensive shock by limiting the need for excessive fluid resuscitation and supporting hemodynamics when shock is refractory to initial fluid boluses. A common accepted definition on when (septic) shock becomes fluid-refractory does not exist. The American Heart Association and American Academy of Pediatrics, as well as the Society of Critical Care Medicine, recommend that after initial resuscitation with isotonic crystalloids, vasoactive agents (epinephrine, norepinephrine) should be started promptly in children with persistent shock to avoid further fluid administration and reduce the risk of fluid overload (5, 67).

*Synopsis:*

For children with the 2 most common **shock** categories (**distributive and hypovolemic**) sufficient volume-therapy using **repetitive fast boluses of 10–20 mL/kg balanced crystalloids** remains the first-line therapy in arterial hypotension.
**Fluid overload** should be **monitored** (using cardiac POCUS) and preferably prevented, especially by **early (<1 h) initiating vasoactive therapy**.

## Vasoactive therapy

Mechanistically, early vasopressor administration stabilizes arterial pressure and improves end-organ perfusion without excessive volume expansion.

Expanding on the comparative efficacy and safety of vasoactive agents in pediatric septic and distributive shock, recent network meta-analyses and clinical trials provide further clarity. Norepinephrine seems to be associated with the lowest mortality among single-agent therapies for pediatric fluid-refractory septic shock, with a trend toward improved outcomes compared to epinephrine and dopamine (68, 69).

In children with fluid-refractory septic shock norepinephrine used as first-line vasoactive agent showed the lowest mortality rate, although the majority of the data was derived from nonrandomized studies. Epinephrine significantly reduced the need for mechanical ventilation and showed a trend for lower mortality when compared to dopamine. The use of two vasoactive agents resulted in lower mortality than the use of a single agent (68). Moreover, early vasopressor use within 1 h of septic shock recognition is independently associated with lower odds of mortality (67).

Dose range, receptor activity, influence on stroke volume, heart frequency, cardiac output, systemic vascular resistance, pulmonary vascular resistance, MAP as well as key adverse effects are summarized in Table 3.

**Table 3:** Comparison of vasoactive drugs.

Vasoactive drug	Dose range [µg/kg/min]	Receptor activity	SV	Heart rate	CO	SVR	PVR	MAP	Key adverse effects	Reference
Epinephrine	0.02–0.1	β1, β2	↑↑	↑	↑	↓/↔	↓/↔	↓/↑/↔	Tachyarrhythmia, Myocardial oxygen consumption↑, Hyperglycemia, Lactat acidosis, Splanchnic hypoperfusion	(4, 19, 68–74)
	>0.1–2	α1 + β1	↑	↑↑	↑	↑↑	↑	↑↑		
Norepinephrine	0.02–0.1	α1 > β1	↑/↔	↔/↑	↑	↑	↑/↔	↑	Hypertension, (Tachy-)Arrhythmia (usually not requiring intervention)	(4, 19, 68, 69, 72, 75–80)
	>0.1-2	α1	↔/(↑)	↔	↑/↔	↑↑↑	↑	↑↑↑		
Milrinone	0.25–0.75	PDE-III inhibition (cAMP↑)	↑↑	↔/↑	↑↑	↓↓↓	↓↓	↔/↓	Hypotension, (Tachy-)Arrhythmia, Thrombocytopenia, Accumulation & Vasoplegia in AKI/CRRT	(14, 46, 57, 68, 78, 95–99)
Dobutamine	2–20	β1 > β2	↑↑	↑	↑↑	↓	↓	↔/↓	(Tachy-)Arrhythmia, Hypotension (due to vasodilation), Myocardial ischemia	(78, 84, 108, 109)
Dopamine	1–5	D1/D2	↔/↑	↔	↔/↑	↓/↔	↔	↓/↔	Tachyarrhythmia, Tissue ischemia, Pituitary function↓	(72–74, 82)
	5–10	β1	↑↑	↑	↔/↑	↔	↔	↔/↑		
	>10	α1 + β1	↑/↔	↑↑	↔/↑	↑↑	↑	↑↑		
Vasopressin	0.0001–0.003 U/kg/min	V1 receptor agonism	↔	↔/↓	↔	↑↑↑	↔/↓	↑↑↑	Peripheral & splanchnic ischemia, Hyponatremia, Urine output↓, Thrombocytopenia	(4, 19, 72, 87, 88, 90–92, 110, 111)

Annotation: Dose-effect relationship as well as effects for every drug may differ between individual patients and disease.

SV, Stroke Volume; SVR, Systemic Vascular Resistance; CO, Cardiac Output; PVR, Pulmonary Vascular Resistance; MAP, Mean Arterial Pressure; AKI, Acute Kidney Injury; CRRT, Continuous Renal Replacement Therapy.

***Epinephrine***'s mechanism of action is dose-dependent, with β-adrenergic effects (inotropy, chronotropy, vasodilation) predominating at lower doses and α-adrenergic vasoconstriction becoming more prominent at higher doses (70). Bolus dosing for acute hypotension in the PICU has been described, with doses of 1–5 μg/kg resulting in significant increases in mean arterial blood pressure and heart rate (71). Safety data indicate that epinephrine is generally well tolerated when titrated carefully (72). Comparative efficacy studies show that epinephrine is superior to dopamine in pediatric septic shock, with lower mortality and improved organ function (73, 74).

***Norepinephrine*** acts primarily as a potent α-1 adrenergic agonist, causing peripheral vasoconstriction and increased systemic vascular resistance, which raises MAP. It also has modest β-1 adrenergic activity, supporting cardiac output without significant chronotropic effects (75, 76). The effect on MAP is dose-dependent, and higher doses may be required in severe shock states (77). Safety profile in children is favorable. Myocardial ischemia and tissue necrosis are theoretical risks, particularly with high doses or extravasation (78). Evidence for efficacy in pediatric arterial hypotension or shock is robust. Norepinephrine reliably increases MAP and urine output in neonates and children with shock, including septic shock, and improves perfusion parameters (77, 79). In neonates, norepinephrine is as effective as dopamine for shock reversal, with fewer tachyarrhythmias and better cerebral oxygenation (80). Direct comparison between epinephrine and norepinephrine in children with septic shock demonstrates similar rates of renal outcomes, but norepinephrine may confer a mortality benefit in children without cardiac dysfunction (69).

***Dopamine*** is a sympathomimetic amine used intravenously to treat arterial hypotension and shock in children. Its mechanism of action is dose-dependent (72). Dopamine increases blood pressure and urine output in children with shock, but response is variable and may be diminished in neonates due to immature norepinephrine stores (81). However, a randomized controlled trial in pediatric septic shock found dopamine was associated with higher mortality and healthcare-associated infection compared to epinephrine (73, 74). Hence, dopamine is used less frequently in current practice due to its variable pharmacodynamic profile and potential adverse effects (4, 19, 82).

***Dobutamine*** is a synthetic catecholamine that acts primarily as a β1-adrenergic agonist, increasing myocardial contractility and cardiac output with modest effects on heart rate and minimal direct effect on peripheral vascular resistance. The safety profile in children is generally favorable for short-term use. Myocardial ischemia is a concern, especially at higher doses or in patients with underlying coronary disease (78). Evidence for efficacy in pediatric shock and hypotension demonstrates that dobutamine increases cardiac index and left ventricular stroke work index, with variable effects on systemic vascular resistance and blood pressure. It may particularly be useful in children with low cardiac output states due to myocardial dysfunction, especially those with cardiogenic shock not complicated by severe hypotension (83, 84).

***Vasopressin*** is a non-catecholamine vasopressor that acts primarily via V1 receptors on vascular smooth muscle, causing vasoconstriction and increased systemic vascular resistance, with additional antidiuretic effects via V2 receptors in the renal collecting ducts. Its mechanism is independent of adrenergic receptors, theoretically making it useful in catecholamine-refractory shock. The pressor effect of intravenous vasopressin reaches its peak within 15 min of starting the infusion, with steady-state plasma concentrations achieved after 30 min of continuous infusion. After stopping the infusion, the pressor effect fades within 20 min. Dose-dependent effects include progressive increases in blood pressure and afterload (85, 86). At higher doses, there is a risk of excessive vasoconstriction and increased myocardial workload, potentially leading to ischemia or organ dysfunction (72). The Society of Critical Care Medicine, in the Surviving Sepsis Campaign International Guidelines for the Management of Septic Shock and Sepsis-Associated Organ Dysfunction in Children, states that vasopressin may be considered as an adjunct in children with septic shock who are not responsive to high-dose catecholamines, but the evidence does not support routine use due to lack of mortality benefit and potential for harm (4, 19). The safety profile of vasopressin in children is characterized by risks of hyponatremia, decreased urine output, increased creatinine, and thrombocytopenia, peripheral and splanchnic ischemia, particularly with higher doses or prolonged infusion (87). Notably, compared with norepinephrine and epinephrine, vasopressin increases SVR without significant effecting PVR—therefore it may especially useful in shock with pulmonary hypertension and/or right heart failure (72, 88, 89). The use of vasopressin should be individualized and considered especially in patients with profound periphal vasoplegia despite high dose vasopressor therapy. In a recent retrospective study including 453 critically ill children with congenital heart disease and sepsis receiving vasopressin as second line vasoactive substance, shorter time interval between noradrenaline and vasopressin initiation was associated with lower PICU mortality (90). Furthermore, anecdotal and survey data from adult international intensivist practice confirm widespread use of vasopressin for refractory vasoplegic shock, with most clinicians initiating (low-dose) vasopressin at norepinephrine doses >0.25–0.5 μg/kg/min, aiming to augment blood pressure and reduce catecholamine exposure (91, 92).

For vasoactive infusions implementing standard concentrations may sigificantly reduce overall medication error rates, especially calculation and preparation errors (93, 94).

*Synopsis:*

**Early use of vasoactive therapy (< 1 h)** in children with high demands of fluid therapy due to shock, before significant fluid overload occurs, should be considered.
**Norepinephrine probably** is a good choice as **first-line vasoactive drug** for assumed distributive and refractory hypovolemic shock states
  - With the **exception** of:
    ▪ Assumed primarly **cardiogenic shock/left ventricular dysfunction:** Low dose **epinephrine** (< 0.05 µg/kg/min) and/or milrinone may be considered.
Dopamine should only be used if norepinephrine and epinephrine are not available.
**Dobutamine** can be considered in cases of **low cardiac output/myocardial dysfunction** as alternative to milrinone.

## Inotropic therapy

Epinephrine, dobutamine, milrinone, and levosimendan are commonly used inotropes in pediatric intensive care, each with distinct pharmacologic profiles, indications, and adverse event risks.

***Epinephrine*** and ***Dobutamine*** are described in the preceding section about *vasoactive therapy*.

***Milrinone*** is a phosphodiesterase III inhibitor with inotropic and vasodilatory (inodilator) properties, enhancing cardiac contractility and reducing systemic and pulmonary vascular resistance. Milrinone may improve cardiac output, although the extent of its direct inotropic effect remains subject to debate. It is widely used for low cardiac output syndrome post-cardiac surgery, heart failure (72, 95, 96). Milrinone may be considered in selected cases of right ventricular dysfunction, particularly when reduction of pulmonary vascular resistance (in cases of pulmonary hypertension) is desired, while careful patient selection is required (57, 97). Its use is widespread but heterogeneous, and it is less arrhythmogenic than catecholamines (46, 95). Due to its vasodilatory properties, milrinone does not consistently increase arterial blood pressure and may require concomitant vasoactive support, using low dose norepinephrine (96). Noticeable there is a significant risk of drug accumulation and vasoplegia due to the vasodilatory effect of milrinone in children with acute kidney injury (AKI) and/or undergoing continuous renal replacement therapy (CRRT). Milrinone is primarily renally excreted, and its clearance is markedly reduced in pediatric patients with AKI, leading to prolonged half-life and elevated serum concentrations, even at standard or reduced dosing. CRRT does not reliably clear milrinone, and pharmacokinetic studies in children show wide inter-individual variability in drug clearance, with frequent concentrations outside the therapeutic range and possible accumulation. Therefore milrinone should only be used in selected cases and with extreme caution in children with AKI and/or CRRT (98, 99).

***Levosimendan*** is a calcium sensitizer and potassium channel opener, increasing myocardial contractility and causing vasodilation. It is used for decompensated heart failure and low cardiac output syndrome post-cardiac surgery. Onset is within about 1 h after the start of infusion, with steady states achieved within 5 h (100). In pediatric patients, levosimendan is rapidly distributed with a distribution half-life of approximately 14 min (101). Importantly, the active metabolite OR-1896 has a half-life of 70–80 h, accounting for the prolonged pharmacodynamic effect lasting at least one week after a 24-hour infusion (100). The transformation of OR-1855 into the active OR-1896 is 3.7-fold slower in neonates/infants compared to adults, resulting in lower active metabolite concentrations in younger patients (102). Adverse effects include hypotension and arrhythmias. The American Heart Association notes its efficacy in improving hemodynamics, but evidence for improved outcomes is limited (95, 103). Although the evidence for improving major clinical outcomes (especially mortality or intensive care unit stay) remain limited, levosimendan produces short-term improvements in cardiac output and hemodynamic stability in children with cardiomyopathy (including those with congenital heart disease or myocarditis), particularly in the setting of acute decompensation or low cardiac output syndrome (95, 103, 104). In septic shock with septic cardiomyopathy levosimendan can improve cardiac function as well, but does not improve survival, and its long half life can contribute to prolonged, sometimes difficult-to-manage vasoplegia (105–107).

*Synopsis:*

Clinical experience and limited evidence suggest the use of **milrinone** and **levosimendan** for reducing **low cardiac output syndrome in critically ill children**, with levosimendan likely conferring a mortality benefit in the post-cardiac surgery population.
**Dobutamine** is widely used but **lacks definitive evidence for superiority**. Though, it is a reasonable alternative to milrinone.
For left or right heart ventricular dysfunction milrinone is a commonly used inotrope, as long as there is no severe kidney injury.
In the setting of **cardiomyopathy** (dilatative cardiomyopathy, myocarditis, low cardiac output syndrome in post cardiac surgery, congential heart disease related) **levosimendan may be preferable**.
In septic shock and low cardiac output, myocarditis or cardiomyopathy with renal failure (acute phase of cardiogenic shock) the use of low-dose epinephrine (0.02–0.05 µg/kg/min) for initial stabilization, with subsequent switch to milrinone or levosimendan represents a therapeutic option.

## Transfusion of erythrocytes

Erythrocyte transfusion in children with shock and/or hypotension is primarily indicated to optimize oxygen delivery (DO₂) when there is evidence of inadequate tissue oxygenation and anemia. In critically ill children, including those with septic shock or myocarditis, transfusion increases oxygen delivery, especially in sepsis, but does not consistently improve cardiac output or survival unless there is severe anemia or ongoing hypoperfusion (112).

In hemorrhagic shock, early transfusion of red blood cells, plasma, and platelets in balanced ratios (e.g., 1:1:1 or 2:1:1) is a key component of management and should not be delayed (113).

In non-hemorrhagic shock, red blood cell transfusion is not a primary intervention for the treatment of hypotension. The most up-to-date evidence and guidelines from the American Association of Blood Banks (AABB), the Society of Critical Care Medicine, and the Pediatric Critical Care Transfusion and Anemia Expertise Initiative recommend a restrictive transfusion threshold of hemoglobin less than 7 g/dL for hemodynamically stable critically ill children without cyanotic heart disease or severe hypoxemia. For children with congenital heart disease, thresholds are individualized: 7 g/dL for biventricular repair, 9 g/dL for single-ventricle palliation, and 7–9 g/dL for uncorrected disease (114, 115).

For pediatric patients with (severe) traumatic brain injury, the adoption of a liberal blood transfusion strategy aiming to maintain hemoglobin concentration above 9 g/dL for is suggested (116).

There is no evidence supporting early transfusion (e.g., hemoglobin <10 g/dL) in catecholamine-refractory shock outside of acute hemorrhage or brain injury. In stabilized septic shock, transfusion above 7 g/dL does not improve outcomes. For unstable shock, transfusion decisions should be guided by clinical signs of hypoperfusion, including decreased ScvO2 values, and not solely by hemoglobin level (4, 114), which makes it an option in these cases.

*Synopsis:*

There is **no evidence supporting early transfusion** (hemoglobin > 7 g/dL) of erythrocytes outside acute hemorrhagic shock or brain injury—hence, in catecholamine-refractory arterial hypotension **erythrocyte transfusions should be guided by clinical signs of hypoperfusion**, including decreased ScvO2 values.
In severe **traumatic brain injury** maintaining **hemoglobin** levels **above 9 g/dL** is suggested.

## Peripheral venous access for initiation of vasoactive therapy

Both epinephrine and norepinephrine can be safely administered via peripheral or intraosseous access, allowing prompt initiation before central access is established (62, 69). Delays in initiating vasoactive support should be avoided, as they may contribute to ongoing hemodynamic instability. Meta-analyses and systematic reviews report a low incidence of local adverse events, such as extravasation or infiltration, with pooled rates between 2%–3% for children, and most events are minor and resolve without long-term sequelae (117, 118). Retrospective cohort studies and prospective observational data confirm that extravasation injuries are rare (1%–2%), typically occur with prolonged infusions (>4 days), and are not associated with tissue necrosis or limb ischemia. Most complications are managed conservatively, and pharmacologic intervention is rarely required (119, 120). Short-term peripheral vasoactive administration is feasible in emergency and prehospital settings, allowing timely initiation of therapy and adherence to guidelines, with dilute preparations recommended to minimize risk (121–123). For epinephrine and norepinephrine a dilution of 20 µg/mL (1 mg in 50 mL NaCl0.9%) with an initial infusion rate of 1/3 mL/kg body weight/hour (∼ 0.1 µg/kg/min) can be suggested.

*Synopsis:*

**Peripheral intravenous administration** of vasoactive therapy especially **norepinephrine and epinephrine** is **safe** for short term use and is **effective** in achieving hemodynamic stabilization.

## Cardiac point of care sonography (cardiac POCUS)

Echocardiography has become a critical tool for guiding hemodynamic therapy in children with shock and/or arterial hypotension, enabling rapid differentiation between shock categories and informing targeted management. Echocardiography can help distinguishing between cardiogenic, obstructive, hypovolemic, and vasoplegic shock (124) by assessing ventricular function, chamber sizes, pericardial effusion, and dynamic flow patterns as well as ruling out pneumothorax. For example, reduced left ventricular function and dilated chambers support cardiogenic shock, while findings such as pericardial tamponade or right ventricular dilation with septal shift indicate obstructive shock and increased pulmonary vascular resistance (66, 125).

In pediatric septic shock, serial echocardiography enables early recognition of myocardial dysfunction and hypovolemia not apparent on clinical assessment, allowing timely initiation of inotropic therapy and reducing shock reversal time, fluid overload, and mortality (126).

Echocardiography can directly influence hemodynamic management in critically ill children, leading to improved clinical outcomes, including shorter durations of mechanical ventilation and vasoactive therapy. By distinguishing between near-normal and depressed cardiac function, echocardiography allowes for more precise tailoring of therapy, avoiding inappropriate use of vasoconstrictors and excessive fluids, which can be associated with worse outcomes (127). These findings underscore the importance of echocardiography in optimizing hemodynamic support and reducing the burden of intensive care interventions in children with shock.

The American Heart Association and American Academy of Pediatrics recommend using echocardiography to assess cardiac function and guide therapy in pediatric shock, including the selection and titration of inotropes and vasopressors based on physiologic findings (5). In the current Surviving Sepsis Campaign International Guidelines for the Management of Sepsis and Septic Shock in Children (2026) cardiac and lung POCUS is suggested to guide resuscitation (19).

**Figure 1:**
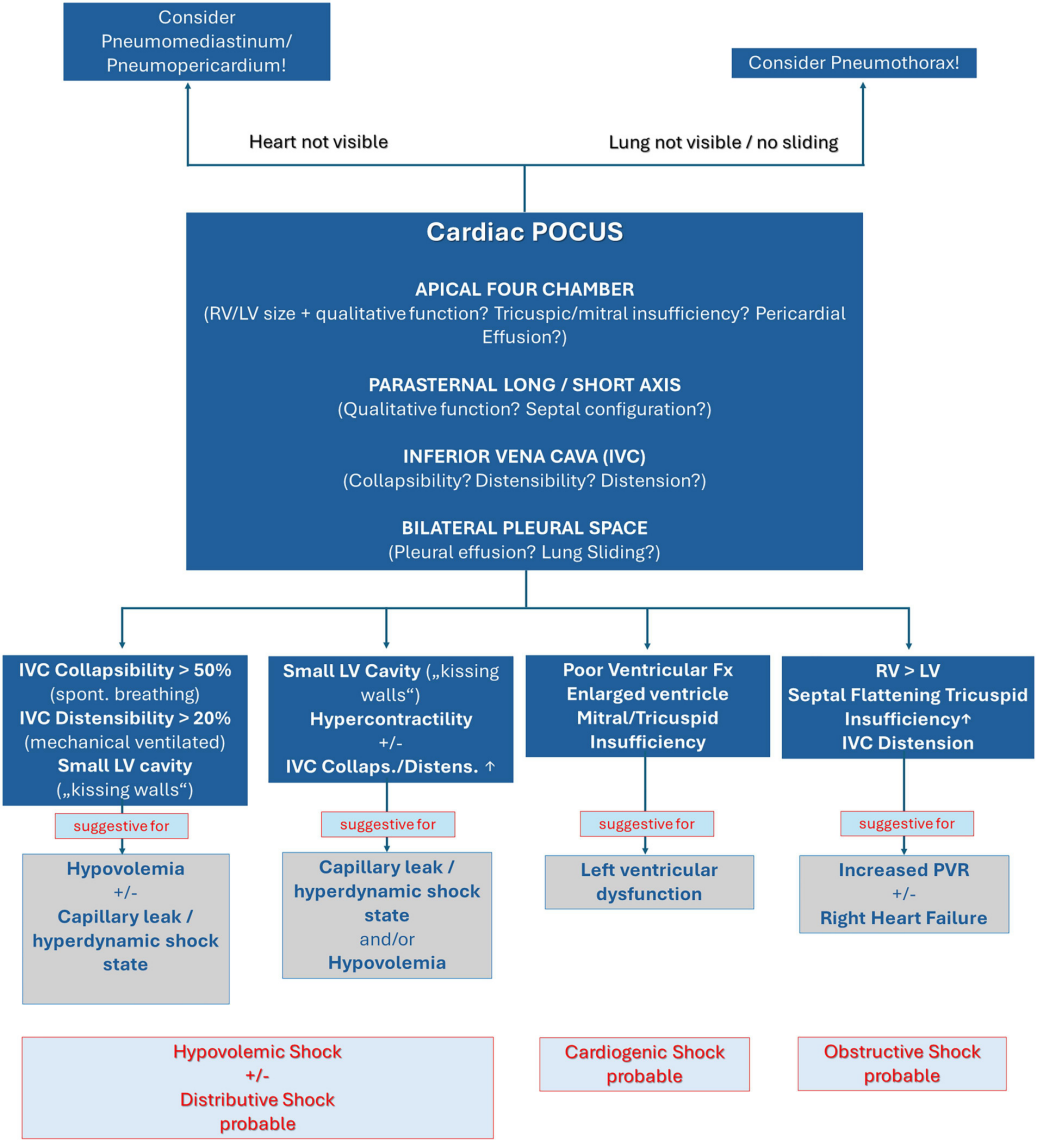
Structured cardiac POCUS-based approach to hemodynamic assessment in critically ill children (19, 66, 124–129). LV, left ventricle; RV, right ventricle; SVR, systemic vascular resistance; PVR, pulmonal vascular resistance; Fx, Function; Collaps., Collabsibility; Distens., Distensibility.

In the majority of pediatric intensive care and emergency cases, a focused point of care echocardiography (cardiac POCUS) protocol is sufficient for most clinical decisions regarding shock management. This protocol should include bilateral pleural space assessment, inferior vena cava evaluation, and standard cardiac views (four-chamber, short/long axis) and enables rapid identification of major causes of shock—such as pericardial effusion, ventricular dysfunction, pulmonary hypertension, and gross volume status—and can guide immediate management in most pediatric intensive care scenarios (128, 129). Figure 1 presents a straightforward framework, which is intended to facilitate rapid, goal-directed assessment in acute care settings and to support clinical decision-making, while acknowledging that findings must always be interpreted within the overall clinical context.

It is still important that clinicians must remain aware of the limitations of cardiac POCUS and seek comprehensive evaluation when necessary (128).

In summary, echocardiography is very helpful, if not essential, for confirming cardiogenic shock, identifying obstructive shock, detecting increased pulmonary vascular resistance, and differentiating between volume and vasoplegia-related etiologies, thereby guiding the necessity for inotropic vs. vasoactive therapy in children with shock.

The schematic overview (Figure 1) summarizes key elements of focused cardiac ultrasound as described in the literature, including assessment of ventricular function, volume status, and pericardial pathology. The figure represents a conceptual synthesis of previously published echocardiographic principles and clinical recommendations and does not include original or unpublished data.

*Synopsis:*

Use **repeated cardiac POCUS** in children with arterial hypotension and suspected shock.
Use a **standard protocol** that includes:
  - **bilateral pleural space assessment** (pleural effusion? lung sliding?)
  - ***inferior vena cava evaluation*** *(Collapsibility Index in spontaneous breathing and Distensibility index in mechanically ventilated patients)*
  - ***standard cardiac views including four/five-chamber and short or long axis views*** *(function? Tricuspic or mitral insufficiency? pericardial effusion? septum deviation?)*

## Options for catecholamine-refractory arterial hypotension

Available guidelines emphasize that refractory (distributive/septic) shock is generally considered when shock persists despite adequate fluid resuscitation and escalating doses of vasoactive agents, but do not endorse a specific numeric cutoff (4).

The Pediatric Organ Dysfunction Information Update Mandate (PODIUM) Consensus Conference defines severe cardiovascular dysfunction in critically ill children as the presence of persistent tachycardia, hypotension, elevated lactate, low central venous oxygen saturation, or echocardiographic evidence of reduced left ventricular ejection fraction, in combination with a high vasoactive-inotropic score, or the need for mechanical circulatory support or cardiopulmonary arrest lasting more than 5 min. These criteria reflect ongoing shock and organ dysfunction despite maximal conventional therapy, including fluids and vasoactive agents (130).

In adults a dosage of norepinephrine > 0.2 µg/kg/min is often deemed “refractory” or as threshold for escalation (131, 132). The dosage of 0.19 µg/kg/min was used in the protocol for the SOAP II trial (133).

Consequently, epinephrine and/or norepinephrine dosages above 0.2 µg/kg/min should trigger considerations for escalation of therapy.

Therapeutic options for catecholamine-refractory shock in children include escalation of vasoactive support, adjunctive corticosteroids, vasopressin, methylene blue, and extracorporeal life support (ECLS/VA-ECMO).

***Hydrocortisone:*** The Society of Critical Care Medicine, in the Surviving Sepsis Campaign International Guidelines for the Management of Septic Shock and Sepsis-Associated Organ Dysfunction in Children, suggests hydrocortisone may be considered in catecholamine-refractory shock, particularly in children at risk for adrenal insufficiency. However, pediatric studies have not demonstrated a mortality benefit (4, 5).

The SHIPSS (Stress Hydrocortisone in Pediatric Septic Shock) trial is currently underway and aims to address many of the unanswered questions associated with hydrocortisone administration. SHIPSS is a multi-institutional, prospective, randomized, double-blinded, placebo-controlled trial examining the use of hydrocortisone for children with fluid- and vasoactive-inotropic-refractory shock (SHIPSS) (134).

Hydrocortisone may hasten shock reversal in some cases—as recently published (135). Although routine use currently is not recommended—adding hydrocortisone in patients with suspected adrenal insufficiency or persistent shock despite fluids and high dose vasopressors seems reasonable and reflects clinical practice.

***Vasopressin:*** The use of vasopressin is described in the section on vasoactive therapy.

***Methylene blue:*** Evidence is limited to case series and small studies, but methylene blue appears to be safe and may increase blood pressure in refractory (distributive) shock states. Methylene blue inhibits guanylate cyclase thereby decreasing the production of cyclic guanylate monophosphate (cGMP). In endothelial cells, decreased cGMP leads to less nitric oxide production, promoting vascular tone (136). Methylene blue seems to increase SVR if measured (64, 137). Still there is no high-quality evidence for improved outcomes, and practice patterns vary widely. Typically a single intravenous bolus dose (0.5–2 mg/kg), repeated boluses or continuous methylene blue infusions (dosage range 0.25–1.5 mg/kg/h for total treatment time of 6–12 h) are used and recommended. Its use may be considered as a rescue therapy in refractory vasoplegic shock (138–140).

***Extracorporeal membrane oxygenation*** *(ECMO*) is considered a rescue therapy for children with shock refractory to conventional management with robust data supporting this practice.

In the context of refractory shock with circulatory failure, veno-arterial (VA) ECMO may be considered to provide hemodynamic support. In contrast, veno-venous (VV) ECMO is primarily used in cases of severe respiratory failure with hypoxemia and preserved cardiac function (4, 19). The choice of modality therefore depends on the predominant underlying pathophysiology.

In distributive (septic) shock, the Society of Critical Care Medicine recommends venoarterial ECMO only after all other treatments have failed, with evidence suggesting survival rates around 59%–65% in pediatric sepsis, and higher rates in neonates (4, 19, 141). High ECMO flows (≥150 mL/kg/min) and central cannulation are associated with lower mortality compared to peripheral cannulation, which carries higher odds of death and more frequent cannulation failures (142, 143). However, peripheral VA-ECMO remains feasible and effective if high flows are achieved early (144). For pediatric cardiogenic shock, VA-ECMO is indicated when medical management fails, providing biventricular and respiratory support. Outcomes depend on patient selection and timing, with survival rates similar to those seen in sepsis (145). In obstructive shock due to pulmonary embolism (PE), VA-ECMO is recommended as a rescue therapy for children in cardiac arrest or severe shock, with registry data showing survival rates of 61% in pediatric PE cases treated with ECMO (146). In children with refractory pulmonary hypertension and right heart failure VA-ECMO may be considered (5, 147).

Clinicians caring for pediatric patients with refractory shock should be aware of the possibility of starting VA-ECMO or transferring patients to a centre with the ability to implement VA-ECMO in these patients.

*Synopsis:*

Epinephrine and/or norepinephrine dosages above 0.2 µg/kg/min should trigger considerations for escalation of therapy.
**Hydrocortisone** (1–2 mg/kg/d) can be considered, especially in cases with suspected or probable adrenal insufficiency and/or persistent shock despite fluids and high dose vasopressors.
**Vasopressin** (0.0001–0.003 U/kg/min) is able to augment blood pressure and reduce catecholamine exposure in catecholamine-refractory hypotension.
**Methylene blue** (bolus of 0.5–2 mg/kg or infusion of 0.25–1.5 mg/kg/h for 6–12 h) appears to be safe and may increase blood pressure in refractory (distributive) shock states.
**ECLS/VA-ECMO** using central cannulation and early achievement of high flows are recommended for refractory distributive/septic, cardiogenic, and obstructive shock.

## Pragmatic clinical approach for arterial hypotension in PICU patients

The algorithms represent a synthesis of individual expert opinion and should be interpreted as dynamic flowcharts rather than prescriptive protocols. They require repeated re-evaluation of the clinical condition (especially the integrated signs of shock), blood pressure readings, and point-of-care echocardiographic findings, and should be applied in the context of clinical judgment and evolving patient-specific factors.

In the management of arterial hypotension in the pediatric intensive care unit, hypotension should invariably be regarded as a clinically significant finding. An initial assessment should address whether the child is in shock and whether factors such as measurement error or oversedation may be contributing to the hypotension.

Concurrent with the initiation of therapy, cardiogenic and obstructive shock should be systematically excluded using clinical assessment, echocardiography, and, where appropriate, additional diagnostic modalities, or, if identified, treated without delay. Until proven otherwise, distributive and/or hypovolemic shock should be presumed.

Sepsis represents an important and not infrequent differential diagnosis and necessitates the prompt initiation of antimicrobial therapy, particularly when a nosocomial infection is suspected.

**Figure 2:**
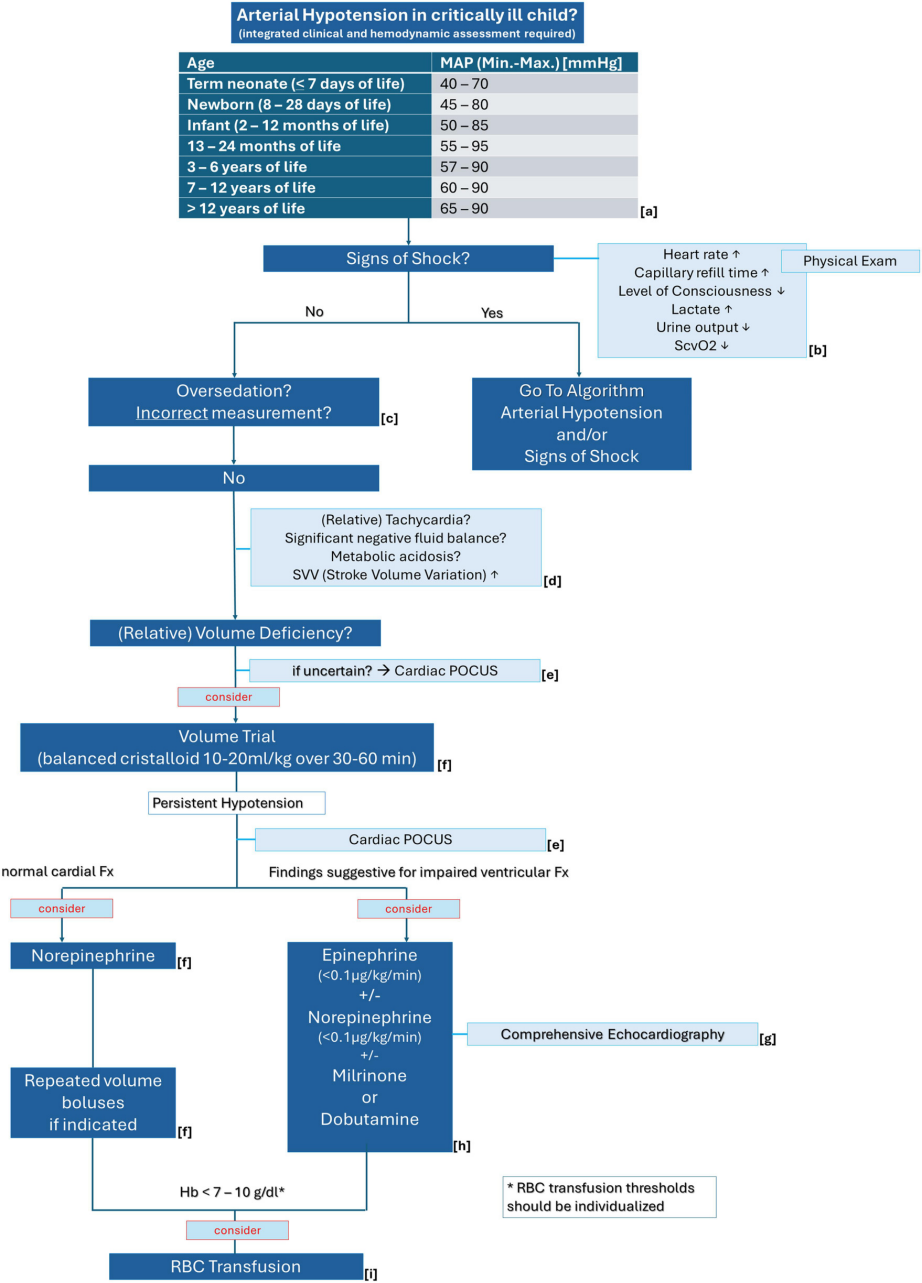
Pragmatic clinical flowchart for initial evaluation of children with arterial hypotension (conceptual synthesis of published evidence and clinical practice patterns). References are included in brackets. MAP, mean arterial pressure; ScvO2, central venous oxygen saturation; Hb, hemoglobin; RBC, red blood cell. References: **(a)** (8, 10, 19, 22, 25, 33); **(b)** (7, 19, 20); **(c)** (42–45); **(d)** (7, 19, 20, 65); **(e)** (19, 66, 124–129); **(f)** (4, 19); **(g)** (128); **(h)** (4, 19, 127); **(i)** (113–115).

**Figure 3:**
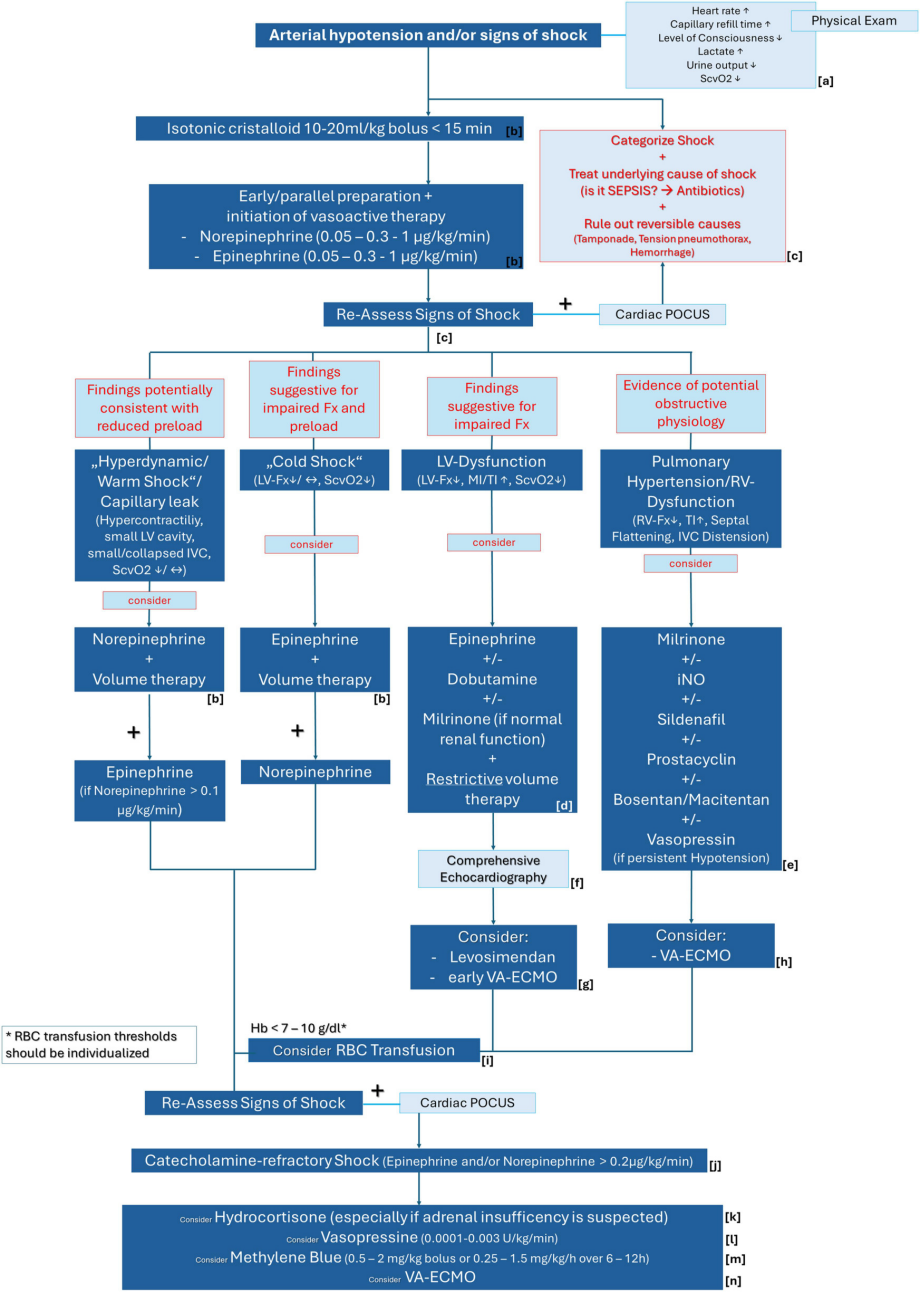
Pragmatic clinical flowchart to arterial hypotension and shock in critically ill children (conceptual synthesis of published evidence and clinical practice patterns). References are included in brackets. ScvO2, central venous oxygen saturation; LV, left ventricle; RV, right ventricle; MI, mitral insufficiency; TI, tricuspid insufficiency; IVC, inferior vena cava; Hb, hemoglobin; RBC, red blood cell; VA-ECMO, venoarterial extracorporal membrane oxygenation. References: **(a)** (7, 19, 20); **(b)** (4, 19); **(c)** (4, 5, 19, 66, 124–129); **(d)** (46, 72, 95, 97, 98); **(e)** (5, 46, 72, 88, 97); **(f)** (128); **(g)** (95, 102–104, 145, 146); **(h)** (5, 147); **(i)** (113–115); **(j)** (19, 121, 130, 148); **(k)** (134, 135); **(l)** (4, 19, 90–92); **(m)** (136–140); **[n]** (4, 19, 141–146).

The provided schematic algorithms (Figures 2, 3) represent a synthesis of published evidence, including international guidelines and clinical studies on pediatric shock and hemodynamic management (see preceding sections). They are intended to provide a structured, bedside-oriented summary of current concepts and do not include original or unpublished data. Given the heterogeneity of available evidence, any structured approach must be interpreted with caution and adapted to the individual clinical scenario.

Many of the recommendations incorporated herein are derived from the evidence base for sepsis and septic shock. However, as discussed above, several of these principles are applicable to other forms of distributive shock associated, in a broader sense, with capillary leak. This consideration is particularly relevant in clinical practice, where patients often require management for presumed sepsis or septic shock in the absence of immediate diagnostic confirmation.

## Future directions

Despite increasing awareness of the clinical relevance of arterial hypotension in critically ill children, important knowledge gaps remain regarding its definition, monitoring, and management. Future research should aim to refine physiologically meaningful thresholds for hypotension that are linked to patient-centered outcomes rather than relying solely on age-based blood pressure values. This is particularly relevant given the heterogeneity of underlying disease processes and the limited generalizability of current evidence.

A key priority is the development and validation of multimodal hemodynamic assessment strategies that integrate clinical examination, laboratory parameters, and advanced monitoring techniques, including point-of-care ultrasound (POCUS). While POCUS has become an increasingly important bedside tool, standardized protocols and pediatric-specific validation studies are still lacking. Prospective studies evaluating the impact of POCUS-guided management on clinical outcomes are needed.

In addition, there is a need for high-quality interventional trials comparing different hemodynamic management strategies, including fluid therapy, vasoactive agents, and inotropic support. Current treatment approaches are largely based on extrapolation from adult data, physiological reasoning, and expert consensus. Pediatric-specific evidence is required to better define optimal therapeutic targets and to guide individualized treatment.

The increasing availability of digital health technologies and real-time data integration offers further opportunities to improve hemodynamic monitoring and decision-making. Future approaches may include the use of predictive analytics and machine learning to identify early signs of hemodynamic deterioration and to support personalized treatment strategies. However, these technologies require careful validation in pediatric populations before widespread clinical implementation.

Finally, efforts should be made to translate existing knowledge into clinically applicable tools that support bedside decision-making. Structured approaches, such as the schematic frameworks presented in this review, may facilitate this process by organizing complex information into accessible formats. Future studies should evaluate their usability, reproducibility, and impact on clinical practice and patient outcomes.

Overall, advancing the care of hypotensive critically ill children will require a combination of improved physiological understanding, pediatric-specific clinical trials, and the development of practical tools that bridge the gap between evidence and bedside application.

## Conclusion

Arterial hypotension in critically ill children represents a complex and multifactorial clinical condition that requires careful interpretation within the broader hemodynamic context. A single blood pressure value alone is often insufficient to guide management, as underlying mechanisms such as hypovolemia, myocardial dysfunction, vasodilation, or obstructive physiology may coexist and evolve over time.

A comprehensive, physiology-based assessment integrating clinical examination, laboratory findings, and, where available, point-of-care ultrasound is essential to support the identification of the predominant hemodynamic state. Such an approach may facilitate more targeted and individualized therapeutic interventions, including fluid resuscitation and vasoactive support, while minimizing the risks associated with non-specific treatment strategies.

Given the limited availability of high-quality pediatric evidence, current management remains largely guided by physiological principles, extrapolation from adult data, and expert consensus. In this context, structured approaches that organize existing knowledge into clinically applicable frameworks may support bedside decision-making, particularly in time-sensitive critical care environments.

The schematic approaches presented in this review are intended as conceptual tools derived from published evidence and established physiological concepts, rather than prescriptive algorithms. Their purpose is to assist clinicians in integrating complex information into a coherent assessment strategy, while emphasizing the need for continuous reassessment and adaptation to the individual patient.

Ultimately, improving outcomes in hypotensive critically ill children will depend on advancing pediatric-specific research, refining hemodynamic monitoring strategies, and further bridging the gap between physiological understanding and clinical practice.
